# Catalysis of Transesterification Reactions by a Self-Assembled Nanosystem

**DOI:** 10.3390/ijms14012011

**Published:** 2013-01-21

**Authors:** Davide Zaramella, Paolo Scrimin, Leonard J. Prins

**Affiliations:** Department of Chemical Sciences, University of Padova, Via Marzolo 1, 35131 Padova, Italy; E-Mail: davide.zaramella@studenti.unipd.it

**Keywords:** supramolecular catalysis, peptides, nanoparticles, self-assembly

## Abstract

Histidine-containing peptides self-assemble on the surface of monolayer protected gold nanoparticles to form a catalytic system for transesterification reactions. Self-assembly is a prerequisite for catalysis, since the isolated peptides do not display catalytic activity by themselves. A series of catalytic peptides and substrates are studied in order to understand the structural parameters that are of relevance to the catalytic efficiency of the system. It is shown that the distance between the His-residue and the anionic tail does not affect the catalytic activity. On the other hand, the catalytic His-residue is sensitive to the chemical nature of the flanking amino acid residues. In particular, the presence of polar Ser-residues causes a significant increase in activity. Finally, kinetic studies of a series of substrates reveal that substrates with a hydrophobic component are very suitable for this catalytic system.

## 1. Introduction

Supramolecular catalysis implies the use of supramolecular chemistry for catalyst development [1–4]. Approaches include the use of recognition motifs to drive active catalyst formation or to change the coordination sphere around a catalytic center [5–7]. Noncovalent interactions are also used to self-assemble molecular capsules to create a confined molecular space, which can affect reactions occurring in the interior [8]. The main advantage of using supramolecular chemistry is that, because relying on self-assembly, the access to the catalyst is very straightforward. This then facilitates the preparation of catalyst libraries and systems of a high complexity. Complex catalytic systems are of interest, because these may manifest features that are common to enzymes, but more difficult to observe in a synthetic system [9,10]. Such phenomena include multivalency, cooperativity and allosterism, which are often fundamental in biological regulatory pathways. Examples of complex self-assembled catalytic systems are micellar systems [11] and self-assembled monolayers on gold nanoparticles [12,13]. The advantage of micelles is their spontaneous formation *in situ*, whereas gold nanoparticle-based systems require a purification step to remove reagents and excess of thiols. On the other hand, the multivalent nature of nanoparticle-based systems is maintained even at low concentrations where micellar systems fall apart. Recently, we have initiated a hybrid approach aimed at combining the positive aspects of both systems by assembling small molecules on the surface of monolayer protected gold nanoparticles [14]. Inspired by contributions of Rotello and co-workers [15–18], we found that anionic peptides and phosphates bound quantitatively to cationic monolayers even at low micromolar concentrations under physiologically relevant conditions [19]. It was shown that complex surfaces could be simply generated by co-assembling different small molecules on the monolayer surface [20].

**Figure 1:**
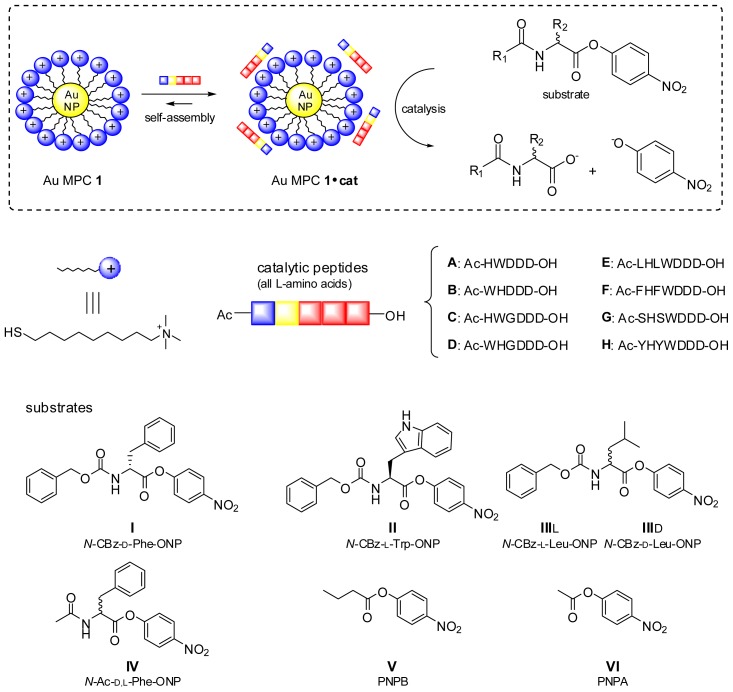
Schematic representation of the catalytic system and structures of catalysts and substrates.

One of our main targets is to use this approach for the formation of complex catalytic systems. Previously, we have shown that the system obtained through the self-assembly of peptide **A** on the monolayer surface of Au MPC **1** catalyses the hydrolysis of substrate **I** (CBz-d-Phe-ONP) (Figure 1) [21]. The rate of hydrolysis is accelerated by nearly two orders of magnitude compared to the background rate (*i.e.*, using the same concentration of peptide **A**, but in the absence of Au MPC **1**). Detailed kinetic studies revealed the following information on the origin of the catalytic effect. First, the linear increase in second-order rate constant as a function of the number of histidines present in an analogous series of peptides revealed that, contrary to what is frequently observed in other multivalent imidazole-based esterolytic catalysts, cooperativity between imidazoles does not appear to play a role in this system. This may be related to the fast exchange kinetics of the systems’ components. This was confirmed by the absence of a bell shaped curve in pH-dependent studies, which is typical for a cooperative mechanism involving a nucleophilic and protonated imidazole [22]. Second, the major origin of catalysis was assigned to the increase in effective concentration resulting from the co-localization of the substrate and the catalyst on the surface of Au MPC **1**. Binding of peptide **A** could be detected directly through fluorescence titration experiments; the affinity of substrate **I** emerged in an indirect manner from the observation that the system displayed Michaelis-Menten saturation behavior with respect to the substrate concentration. Finally, apart from bringing substrate and catalyst in close proximity, the cationic monolayer played an additional role of causing a local higher pH, thus causing an increase of the concentration of deprotonated imidazole, which is the catalytic species. The latter emerged from the nonlinear decrease in rate upon diluting the concentration of **A** on the surface. These experiments provided insight in the functioning of this catalytic system, but were based only on a single couple of catalyst and substrate. Here, we extend our studies towards other catalytic peptides and substrates with the main goal of determining the scope of the system. In particular, we were interested to determine the effect of structural changes in both catalyst and substrate on the catalytic efficiency. The outcome could then confirm and further improve our understanding of the system and set its boundaries.

## 2. Results and Discussion

### 2.1. Structural Modifications in the Catalytic Peptide

Our first interest was in investigating to which extent the catalytic efficiency of peptide **A** was affected by small structural changes, in particular regarding the distance between the anionic tail and the His-residue. For that reason, two analogues of **A** were synthesized with the His-residue immediately attached to the DDD–fragment (**B**) or separated by two amino acids (**C**). In all sequences, also, a Trp-residue was added as a fluorescent probe to facilitate detection of peptide binding to the nanoparticles. Peptide **D** was added to study whether the Trp-spacer in the original peptide **A** was of importance. All peptides were synthesized on solid support using Fmoc-chemistry and, after cleavage, purified by RP-HPLC and characterized by UPLC and MS (see Supporting Information).

**Figure 2:**
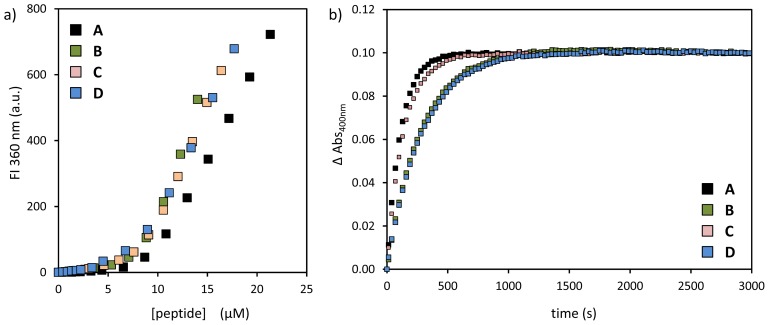
(**a**) Tryptophan fluorescence intensity at 360 nm as a function of the concentration of peptides **A**–**D** in the presence of Au MPC **1** ([headgroup] = 60 ± 5 μM). Conditions: pH 7.0; [HEPES] = 10 mM; H_2_O/CH_3_CN = 90:10; T = 37 °C; (**b**) Changes in the absorbance at 400 nm upon the addition of substrate **I** to a solution of Au MPC **1** and either one of the peptides **A**–**D**. Conditions: Au MPC **1** [headgroup] = 60 ± 5 μM; [**A**] = 4.8 μM, [**B**] = 4.3 μM, [**C**] = 4.3 μM, [**D**] = 3.9 μM; [HEPES] = 10 mM; H_2_O/CH_3_CN = 90:10; pH 7.0; T = 37 °C.

The complexation of peptides **A**–**D** on the monolayer surface of Au MPC **1** was studied by fluorescence titrations as described before under identical experimental conditions ([R-N(CH_3_)_3_^+^] = 60 ± 10 μM; H_2_O:CH_3_CN = 90:10; [HEPES] = 10 mM; pH = 7.0; T = 37 °C) [21]. Also previously studied, peptide **A** was added in order to have a data set performed with the same batch of nanoparticles [21]. The resulting binding isotherms (Figure 2a) reveal that all peptides bind the cationic monolayer under saturation conditions. Surface saturation concentrations (SSCs) were determined as reported before and are listed in Table 1 [21]. All peptides have similar SSCs in the range between 7.8 (**D**) and 9.5 (**A**) [23]. Subsequently, kinetic studies were performed to determine the efficiency of the catalytic systems in accelerating the hydrolysis of the substrate **I** (Figure 2b). Kinetic experiments were performed by adding the substrate (10 μM) to a solution of Au MPC **1** and the peptides **A**–**D** in a H_2_O:CH_3_CN (9:1) mixture buffered at pH 7.0 at 37 °C. Peptides **A**–**D** were present at concentrations corresponding to 50% of their respective surface saturation concentrations, since we had previously shown that these conditions were favorable for catalysis (see above) [21]. From the kinetic profiles pseudo-first order rate constants, *k*_obs_, were obtained by fitting the experimental data to an appropriate model. In order to permit a direct comparison between the different peptides, the *k*_obs_-values were corrected for the peptide-concentration to yield the second order rates constants, *k*_2_ (Table 1). It is noted that in the absence of Au MPC **1**, none of the peptides displayed any significant activity (data not shown). Analysis of the data shows that the distance between the His-residue and the anionic tail is not crucial with peptides **A** and **C**, having nearly identical rate constants (*k*_2,_**_A_** = 2.1 × 10^3^ M^−1^·s^−1^ and *k*_2,_**_C_** = 1.7 × 10^3^ M^−1^·s^−1^). In fact, peptide **B**, which does not even have a spacer between the His-residue and the DDD-tail, has a second-order rate constant that is the lowest (*k*_2,_**_B_** = 0.8 × 10^3^ M^−1^·s^−1^). Rather, the sequence order of the catalytic peptide seems of more importance, *i.e.*, the presence of a Trp-residue preceding the His-residue (HisTrp as in **A** and **C** compared to TrpHis in **B** and **D**). Previous studies have recently demonstrated that hydrophobic units (such as Trp) present in the probes can be involved in binding to the monolayer, because of interactions with the hydrophobic interior part of the monolayer [24,25]. The obtained data suggest that such an interaction may favor catalytic activity by affecting the relative position of the His-residue compared to the cationic head groups. It is noted, though, that the observed effect on catalytic activity is relatively small.

**Figure 3:**
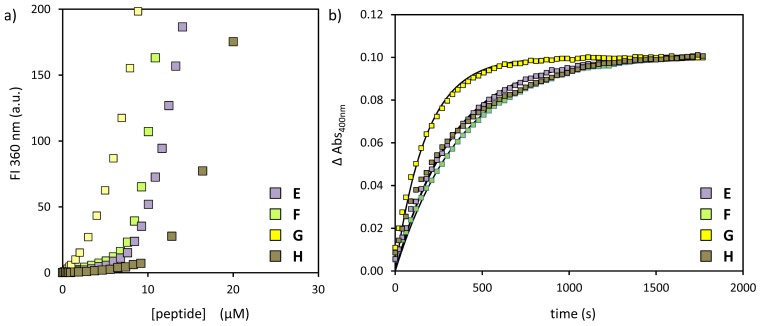
(**a**) Tryptophan fluorescence intensity at 360 nm as a function of the concentration of peptides **A**–**D** in the presence of Au MPC **1** ([headgroup] = 60 ± 5 μM). Conditions: pH 7.0; [HEPES] = 10 mM; H_2_O/CH_3_CN = 90:10; T = 37 °C; (**b**) Changes in the absorbance at 400 nm upon the addition of **I** (10 μM) to a solution of Au MPC **1** and either one of the peptides **E**–**H**. Conditions: Au MPC **1** [headgroup] = 60 ± 5 μM; [**E**] = 4.85 μM, [**F**] = 5.30 μM, [**G**] = 4.25 μM, [**H**] = 7.95 μM; [HEPES] = 10 mM; H_2_O/CH_3_CN = 90:10; pH 7.0; T = 37 °C.

We next proceeded with the synthesis and study of a series of peptides aimed at determining whether the catalytic activity of the His-residue could be affected by flanking residues. For that purpose, peptides **E**–**H** were synthesized with the consensus sequence Ac-*X*H*X*WDDD-OH (Figure 1). The His-residue was positioned between Leu (**E**)-, Phe (**F**)-, Ser (**G**)- and Tyr (**H**)-residues in order to explore different local chemical environments. All peptides were synthesized on solid support using Fmoc-chemistry and, after cleavage, purified by RP-HPLC and characterized by UPLC and MS (see Supporting Information). As before, the complexation of peptides **E**–**H** on the monolayer surface of Au MPC **1** was studied by fluorescence titrations (Figure 3a). From the obtained curves, it is immediately evident that the chemical nature of the catalytic tail significantly affects the SSC (Table 2). The highest SSC (15.9 ± 0.2 μM) is observed for peptide **H** containing two Tyr-residues. Peptides **E** and **F** have intermediate SSC values of 8.6 ± 0.2 μM and 9.1 ± 0.2 μM, respectively, whereas peptide **G** with polar Ser-residues has a significantly lower SSC (4.4 ± 0.2 μM ). The shallow curve observed for peptide **G** is a sign of the lower affinity of this peptide for the monolayer surface. Kinetic experiments were performed as described earlier using substrate **I** (Figure 3b), and the results are given in Table 2. The obtained data (*k*^2^-value, *i.e.*, corrected for the peptide concentration) clearly show an effect of the peptide sequence on catalytic activity. In particular, a higher activity is observed for the His-residue in peptide **G** surrounded by Ser-residues. Tentatively, this might be explained by an enhanced stabilization of the polar transition state of the reaction by the polar peptide sequence. One could argue that the lower SSC of peptide **G** causes an enhanced activity of the system, because of a lesser inhibition of the intrinsic catalytic activity of Au MPC **1**. However, the intrinsic activity of Au MPC **1** (*k*_obs_ = 1.0 × 10^−3^ s^−1^, see below) is too low to explain the observed difference.

### 2.2. Substrate Variation

A series of substrates were tested in order to assess the relation between substrate structure and catalytic efficiency. Apart from the reference substrate used so far (**I**), substrates **II**–**VI** were selected. All substrates are *p*-nitrophenyl activated carboxylic acids, which ensures reactivity and permits a straightforward measurement of the reaction kinetics by UV-Vis spectroscopy. In addition, the transesterification reaction of **II** can also be studied by fluorescence, since hydrolysis removes the *p*-nitrophenyl group, which is a quencher of the Trp-fluorescence. Both substrates **II** and **III** provide information on the importance of the side chain substituent. In addition, both the d- and l-enantiomers of **III** (**III**_L_ and **III**_D_) were studied in order to detect possible enantioselectivity. Substrate **IV** carries an acetyl group instead of the CBz-group and, finally, substrates **V** and **VI** are simple, non-peptidic substrates of different polarity. Catalytic peptide **A** was used as the reference catalyst.

**Figure 4:**
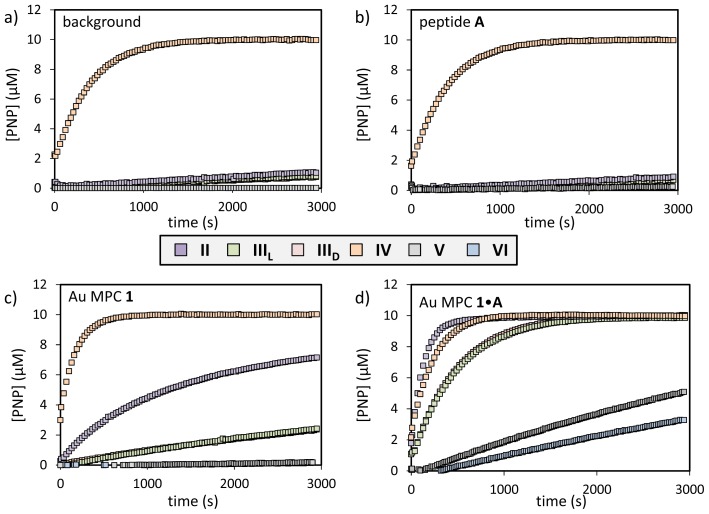
Amount of released *p*-nitrophenol as a function of time upon the addition of substrates **II**–**VI** to solutions of (**a**) background; (**b**) peptide **A** (4.8 μM); (**c**) Au MPC **1** ([headgroup] = 60 ± 5 μM and (**d**) Au MPC **1**•**A** ([headgroup] = 60 ± 5 μM; [**A**] = 4.8 μM). Other conditions: [HEPES] = 10 mM; H_2_O/CH_3_CN = 90:10; pH 7.0; T = 37 °C.

First, the background hydrolysis rate was measured for each new substrate by measuring the increase in absorption at 400 nm as a function of time of a solution of the respective substrate (10 μM) in a 9:1 H_2_O:CH_3_CN mixture buffered at pH = 7.0 at 37 °C ([HEPES] = 10 mM) (Figure 4a). The concentration of 10 μM is set to avoid solubility problems. None of the substrates showed significant degrees of hydrolysis over a period of 1 hour, except for substrate **IV**, which was completely hydrolyzed within 30 min (*k*_obs_ = 2.5 × 10^−3^·s^−1^). For the other substrates, first-order rate constants were determined by fixing the known end value of 10 μM (Table 3). The addition of catalyst **A** (4.8 μM) without nanoparticles did not significantly affect the hydrolysis rate of the substrates (Figure 4b). On the other hand, similar as in our previous studies [21], in all cases the addition of Au MPC **1** by itself caused a rate acceleration (except for substrates **V** and **VI**), but this intrinsic contribution could be suppressed by saturating the monolayer surface with catalytically inert peptide Ac-WDDD-OH (11 μM) (Table 3) (Figure 4c). The latter situation serves as a reference system to assess the actual rate acceleration originating from the catalytic peptide **A** on the surface of Au MPC **1** (Figure 4d). The addition of catalyst **A** (4.8 μM corresponding to 50% of the SSC) in the presence of Au MPC **1** resulted for all substrates in a significant rate acceleration (Table 3).

First of all, the data obtained for substrate **IV** show that the CBz-protecting group serves to prevent a rapid auto-hydrolysis of the substrate. However, even for substrate **IV**, a catalytic effect of Au MPC **1**•**A** is observed (*k*_obs,_**_1•A_** = 4.2 × 10^−3^·s^−1^ against *k*_obs_,**_1•WDDD_** = 2.5 × 10^−3^·s^−1^), although relatively small. The observation that the strongest rate enhancement is observed for substrates **I**–**III** suggests that the CBz-group plays a role in binding of the substrate to the monolayer. Indeed, the rate acceleration observed for substrate **IV** lacking the CBz-group is much lower even if just Au MPC **1** is added. Although structurally very simple, the small substrates **V** and **VI** are interesting, because their hydrolysis is not catalyzed at all by Au MPC **1** alone, suggesting a much lower affinity of these substrates for the monolayer. In the presence of the catalytic system, Au MPC **1**•**A**, a rate acceleration is observed, but the absolute rates are still much lower compared to the larger apolar substrates **I**–**IV**. Nonetheless, also in this case, the effect is stronger on substrate **VI**, which has a larger hydrophobic tail compared to **III**. In summary, the catalytic activity of Au MPC **1**•**A** is not restricted to substrate **I**, but appears to be general for substrates with a significant hydrophobic component. The identical rate constants obtained for enantiomeric substrates **III**_L_ and **III**_D_ show that the system is not enantioselective at all.

**Figure 5:**
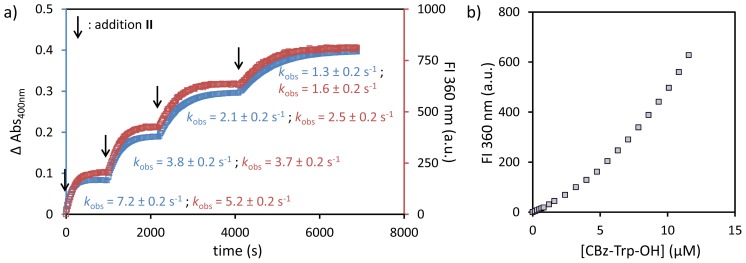
(**a**) Changes in the absorbance at 400 nm (blue) and the tryptophane fluorescence intensity at 360 nm (red) upon four consecutive additions of substrate **II** (4 × 10 μM) to a solution of Au MPC **1**•**A**. Conditions: Au MPC **1** [headgroup] = 60 ± 5 μM; [**A**] = 4.8 μM; [HEPES] = 10 mM; H_2_O/CH_3_CN = 90:10; pH 7.0; T = 37 °C. λ_ex_= 280 nm; slits 5/10. (**b**) Tryptophan fluorescence intensity at 360 nm as a function of the concentration of CBz-(L)-Trp-OH in the presence of Au MPC **1** ([headgroup] = 60 ± 5 μM). Conditions: pH 7.0; [HEPES] = 10 mM; H_2_O/CH_3_CN = 90:10; T = 37 °C.

More information on the catalytic system was obtained from a detailed study of substrate **II**. As stated before, the hydrolysis kinetics of substrate **II** can be followed simultaneously by UV-Vis and fluorescence spectroscopy, because hydrolysis results in the formation of a chromogenic (*p*-nitrophenolate) and fluorogenic (CBz-Tryp-OH) product. Thus, the hydrolysis of substrate **II** was followed simultaneously by monitoring the fluorescence intensity at 360 nm (CBz-Tryp-OH) and the UV absorption at 400 nm (*p*-nitrophenol). Four catalytic cycles were performed by sequentially adding 10 μM of substrate **II**. As illustrated in Figure 5a, the kinetic profiles give a perfect match, which, together with the observed turnover of the system, is definitive proof of the catalytic activity of Au MPC **1**•**A**. The slightly lower rate constants obtained from the fluorescence trace may be a result of product binding to the monolayer. In fact, it is observed that the rate constants show a constant drop after each cycle. This is indicative of the accumulation of an inhibitor in the system, which disfavors formation of the catalytic system composed of Au MPC **1**, catalytic peptide **A** and substrate **II**. The most likely candidate is the carboxylic acid, CBz-Tryp-OH, as it combines a negative charge with a hydrophobic unit. Indeed, a direct fluorescence titration of CBz-Tryp-OH to Au MPC **1** yielded a curve that deviates from linearity in the 0–10 μM concentration regime, which indicates some weak affinity for the monolayer (Figure 5b). Most likely, the accumulation of the reaction product CBz-Tryp-OH in the system affects the equilibrium between Au MPC **1** and substrate **II**, as the latter is the weakly bound component in the system.

## 3. Conclusions

The data presented here give a broader insight into the functioning of the self-assembled catalytic system that we have reported earlier. In particular, a series of catalytic peptides and substrates give insight into the structural parameters that are relevant to the catalytic performance. Whereas the distance between His-residue and the anionic tail is of less relevance, the sequence order induces some changes in the activity. Of more importance is that the activity of the catalytic His-residue can be modulated through the insertion of flanking residues. Compared to apolar residues, the presence of two polar Ser-residues as neighboring units causes a significant increase in rate. This is an important result, as it is another example of controlling the properties of a complex supramolecular catalytic system simply through small structural changes in the building blocks. The kinetic studies of a series of substrates reveal that, in particular, substrates with a hydrophobic component are very suitable for this catalytic system. This confirms our initial assumption that hydrophobic interactions with the hydrophobic part of the monolayer drive substrate binding, which causes an increase in effective concentration. The simultaneous study of the hydrolysis of substrate **II** by UV-Vis and fluorescence spectroscopy gave unequivocal evidence of the catalytic activity of our system. Binding studies of the reaction product revealed that competition between the carboxylate and the substrate are probably at the origin of the reduced efficiency of the system after multiple turnovers. On the other hand, so far, we have not yet been able to detect enantioselective hydrolysis, which remains a future challenge for this system.

## Figures and Tables

**Table 1:** Surface saturation concentrations (SSC), observed rate constants, *k*_obs_, and second order rate constants, *k*_2_, for peptides **A**–**D**, as determined from Figure 2.

peptide	SSC (μM)	*k*_obs_ (×10^−3^·s^−1^)	*k*_2_ (×10^3^ L·mol^−1^·s^−1^)
**A**	9.5 ± 0.3	8.9	2.1
**B**	8.6 ± 0.2	3.6	0.8
**C**	8.5 ± 0.2	7.1	1.7
**D**	7.8 ± 0.2	3.5	0.9

**Table 2:** Surface saturation concentrations (SSC), observed rate constants, *k*_obs_ and second order rate constants *k*_2_ for peptides **E**–**H** as determined from Figure 2.

peptide	SSC (μM)	*k*_obs_ (×10^−3^·s^−1^)	*k*_2_ (×10^3^ L·mol^−1^·s^−1^)
**E**	8.6 ± 0.3	3.0	620
**F**	9.1 ± 0.2	2.6	490
**G**	4.4 ± 0.2	5.6	1310
**H**	15.9 ± 0.2	3.0	380

**Table 3:** Rate constants (*k*_obs_) for substrates **I**–**IV**. Only the pseudo-first order rate constants are reported, since all studies were performed at a constant concentration of **A**.

substrate	background (×10^−3^·s^−1^)	catalyst A (×10^−3^·s^−1^)	Au MPC 1 (×10^−3^·s^−1^)	Au MPC 1•A (×10^−3^·s^−1^)	Au MPC 1•WDDD (×10^−3^·s^−1^)	Ratio a
**I**	0.05	0.05	1.0	8.9	0.3	30
**II**	0.01	0.03	0.9	7.2	0.4	18
**III_L_**	0.05	0.04	0.2	2.0	0.1	20
**III_D_**	0.05	0.04	0.2	2.0	0.1	20
**IV**	2.5	2.5	5.5	4.2	2.5	1.7
**V**	0.01	-	0.01	0.1	0.01	10^b^
**VI**	0.01	0.01	0.01	0.2	0.01	20^b^

a The ratio refers to the observed rate acceleration between the catalytic system Au MPC **1**•**A** and Au MPC **1**•WDDD (*i.e.*, columns 5 and 6, respectively);

b This ratio is mainly determined by the very low rate constant determined for Au MPC **1**•WDDD.

## Supporting Information

Supporting information regarding instrumentation, experimental protocols and characterization data for peptides **B**–**H** is available.

## Supplementary Files

Supplementary File 1 Supporting Information (PDF, 151 KB)
