# Supplementary material for: Catalysis of Transesterification Reactions by a Self-Assembled Nanosystem
Source: Int J Mol Sci. 2013 Jan 21;14(1):2011–21. doi: 10.3390/ijms14012011 (PMC3565362; doi:10.3390/ijms14012011)
Supplement: Supplementary File 1 — Supporting Information (PDF, 151 KB) [file ijms-14-02011-s001.pdf]

Supporting Information

1. Instrumentation and Reagents

Peptide synthesis was performed on a Zinsser Sophas synthesizer starting from Wang resin (0.8 mmol/g; 100–200 mesh) using standard Fmoc-chemistry. All commercially available reagents and solvents were used without further purification.

HPLC purifications were performed on a preparative HPLC Shimazu LC-8A equipped with a Shimazu SPD-20A UV detector. The column used for separation was a Jupiter Proteo 4 μ 90 Å 250 × 21.2 mm, flow: 17 mL/min, eluents: H_2O + 0.1% HCOOH (A), CH_3CN + 0.1% HCOOH (B), gradient: 0–30 min 5%–35% B, λ_{det} = 226 nm. The chromatographic column used for separations in UHPLC analysis was a Zorbax RRHP Eclipse Plus C18 2.1 × 100 mm, 1.8 μm, flow 0.2 mL/min, eluents: H_2O + 0.1% HCOOH (A), CH_3CN + 0.1% HCOOH (B), gradient: 0–5 min 10%–90% B. Aqueous phases were concentrated at reduced pressure using a Genevac EZ-2 Plus centrifuge.

The UPLC/MS analysis were performed using an Agilent 1290 Infinity UPLC equipped with diode array and ESI-MS detector. The chromatographic column used was an Agilent RRHD Zorbax Eclipse Plus C18 (2.1 × 150 mm 1.8 micron), flow 0.2 mL/min, from 2% to 52% CAN + 0.1% HCOOH in 5 min. High resolution mass-spectra were recorded on a Mariner ESI-TOF spectrometer (Perceptive BioSystems) in positive ion mode, as indicated.

Kinetic experiments were performed on a Varian Cary 100 UV/Vis spectrophotometer equipped with thermostatted multiple cell holders.

Fluorescence spectra were recorded on a Varian Cary Eclipse Fluorescence spectrophotometer equipped with a thermostatted cell holder.

2. Protocols

All binding studies and kinetic studies were performed in an analogous manner, as reported before by Zaramella, *et al.* [21].

3. Synthesis and Characterization of B–H

The synthesis of peptides **B**–**H** was performed on a Zinsser Sophas synthesizer starting from Wang resin (0.8 mmol/g; 100–200 mesh) using standard Fmoc-chemistry using DIC/HOBt as coupling agents. After TFA cleavage, the peptides were precipitated using Et_2O and purified with preparative RP-HPLC (Jupiter Proteo 4 μ, 90 Å, 250 × 21.2 mm, flow: 17 mL/min, eluents: H_2O + 0.1% HCOOH (A), CH_3CN + 0.1% HCOOH (B), gradient: 0–30 min 5%–35% B, λ_{det} = 226 nm). The purified products were analyzed with UPLC/MS (Agilent Zorbax RRHD Eclipse C18, 1.8 μ, 2.1 × 150 mm, flow: 0.2 mL/min (peptides **B**–**D**) and 0.4 mL/min (peptides **E**–**H**), eluents: H_2O + 0.1% HCOOH (A), CH_3CN + 0.1% HCOOH (B), gradient: 0–5 min 2%–52% B, T = 40 °C, λ_{det} = 240–350 nm).

Figure S1. Ultra High Performance Liquid Chromatography (UHPLC) chromatogram of Ac-WHDDD-OH (**B**). Gradient 2%–52% of CH_3CN in 5 min with 0.1% HCOOH. Flow 0.2 mL/min. λ= 220 nm.

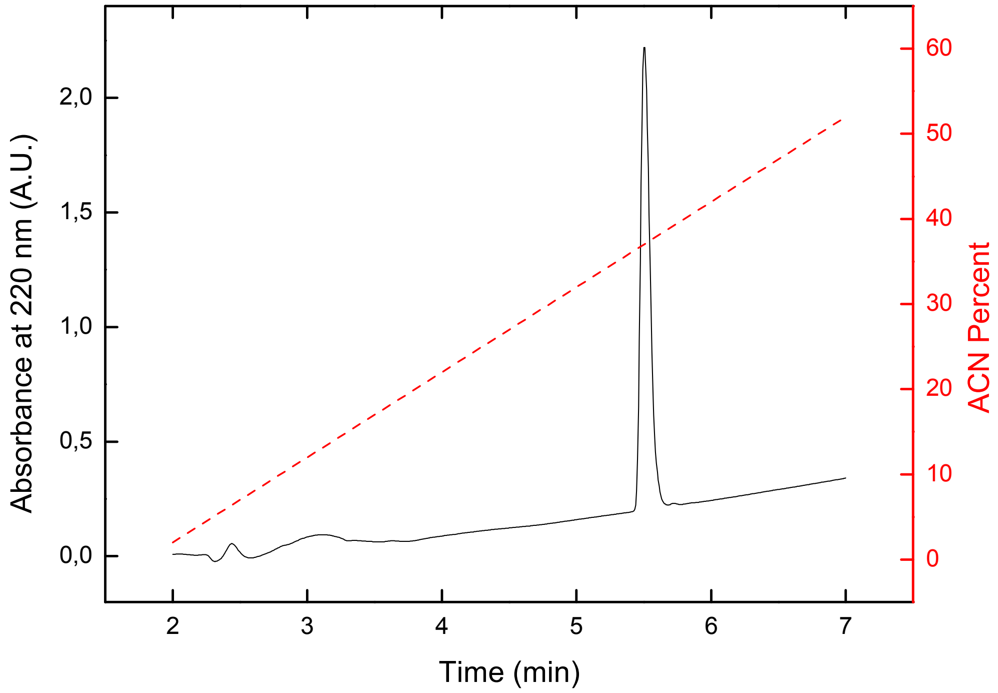

Figure S2. *m*/*z* spectrum of Ac-WHDDD-OH (**B**); theoretical MW_{mono} = 728.229 Da.

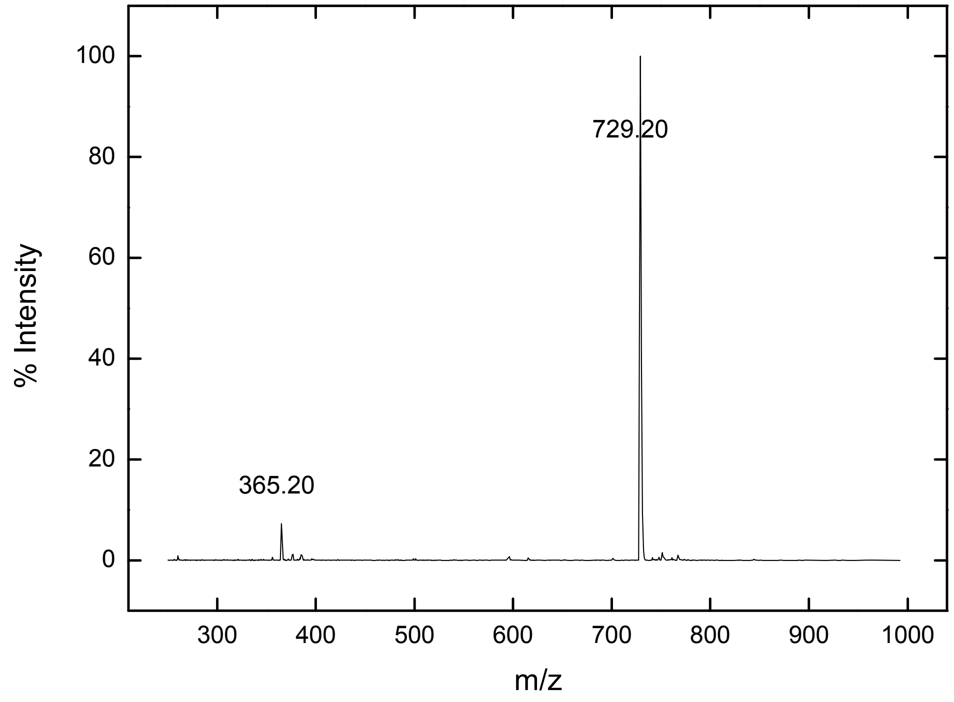

Figure S3. UHPLC chromatogram of Ac-HWGDDD-OH (**C**). Gradient 2%–52% of ACN in 5 min with 0.1% HCOOH. Flow 0.2 mL/min. λ= 220 nm.

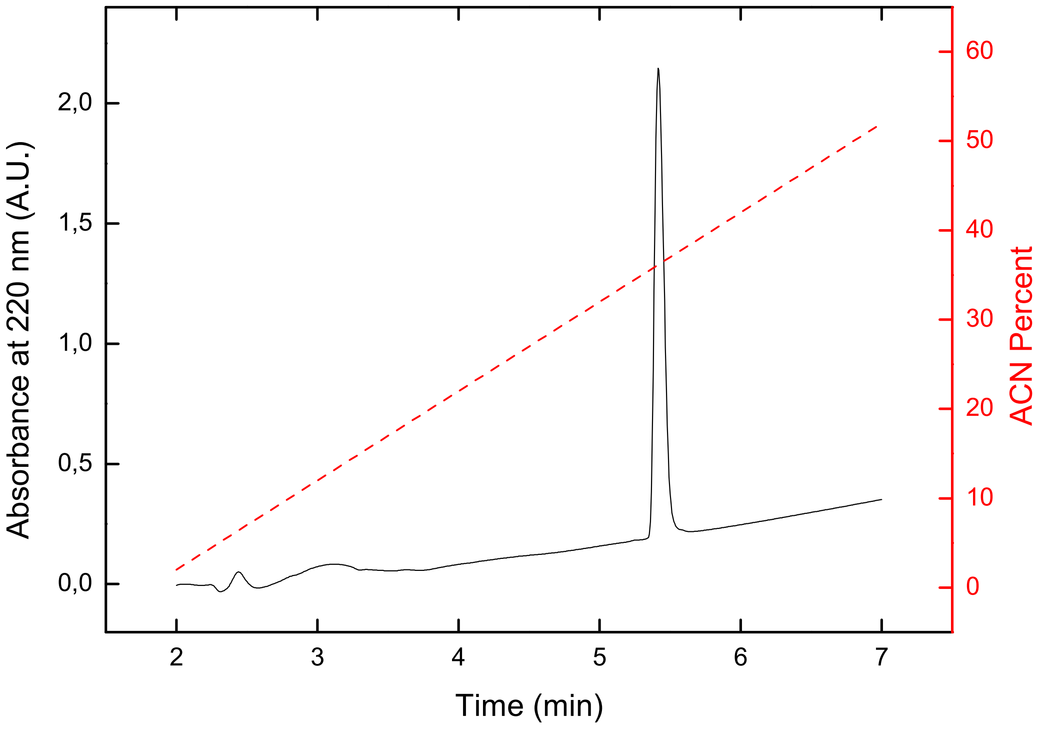

Figure S4. *m*/*z* spectrum of Ac-HWGDDD-OH (**C**): theoretical MW_{mono} = 785.251 Da.

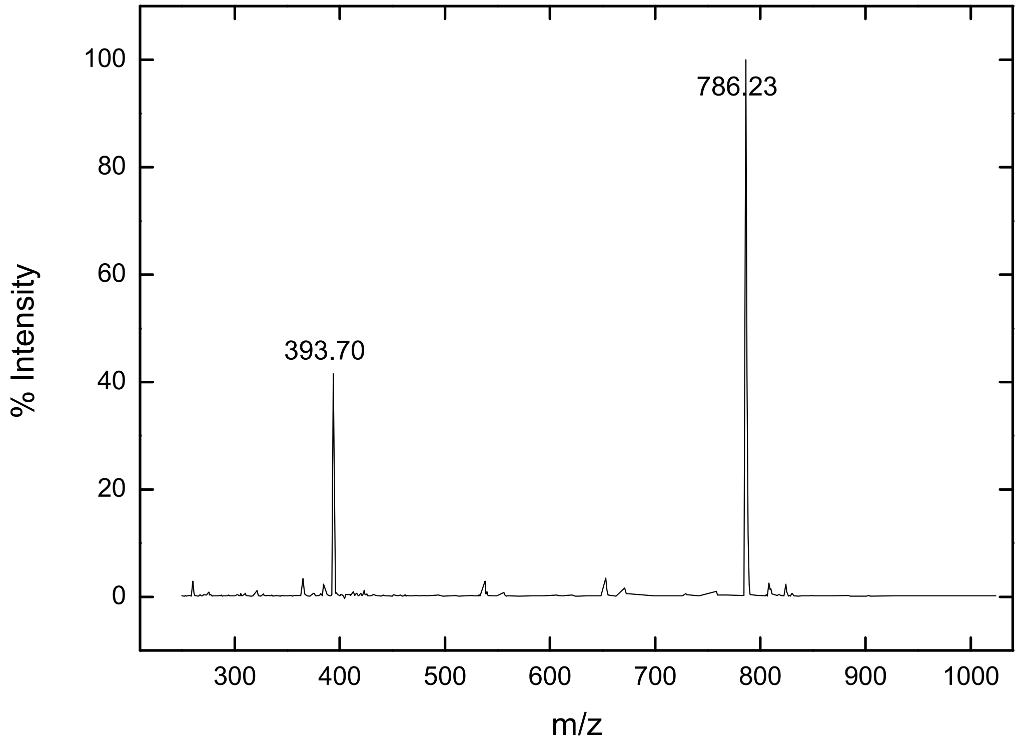

Figure S5. UHPLC chromatogram of Ac-WHGDDD-OH (**D**). Gradient 2%–52% of ACN in 5 min with 0.1% HCOOH. Flow 0.2 mL/min. λ = 220 nm.

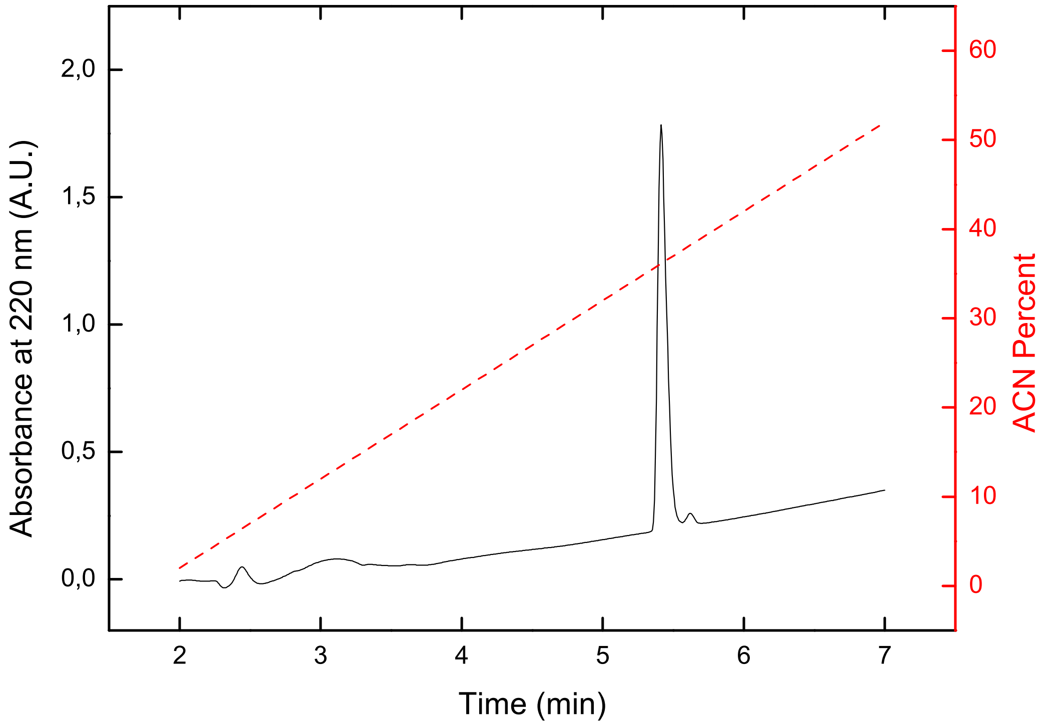

Figure S6. *m*/*z* spectrum of Ac-WHGDDD-OH (**D**): theoretical MW_{mono} = 785.251 Da.

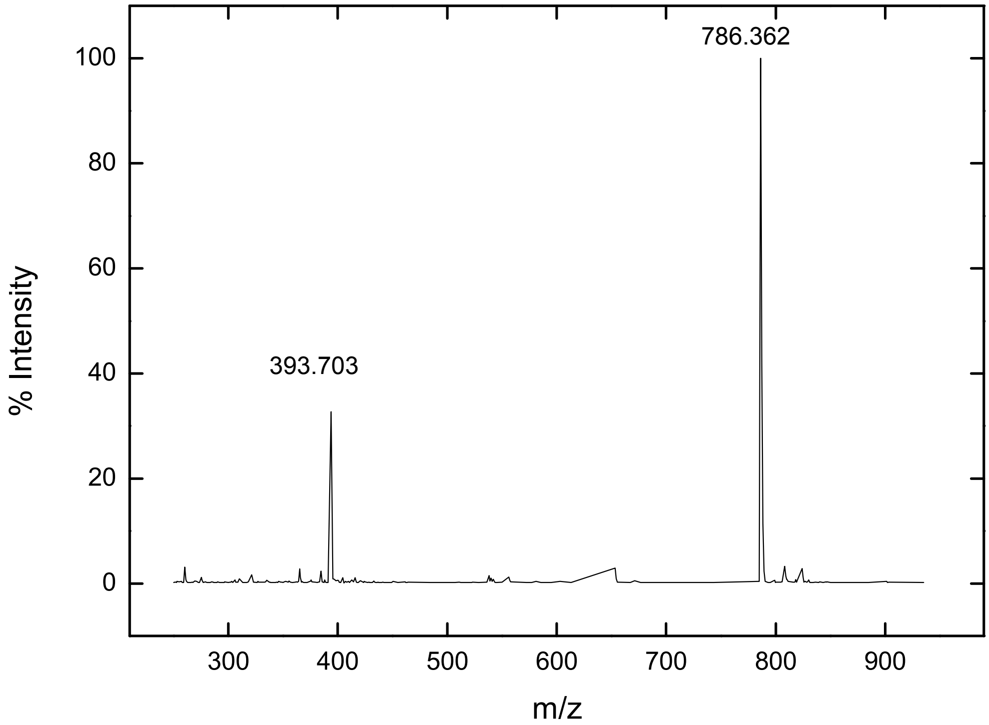

Figure S7. UHPLC chromatogram of Ac-FHFWDDD-OH (**E**). Gradient 2%–52% of ACN in 5 min with 0.1% HCOOH. Flow 0.4 mL/min. λ= 220 nm.

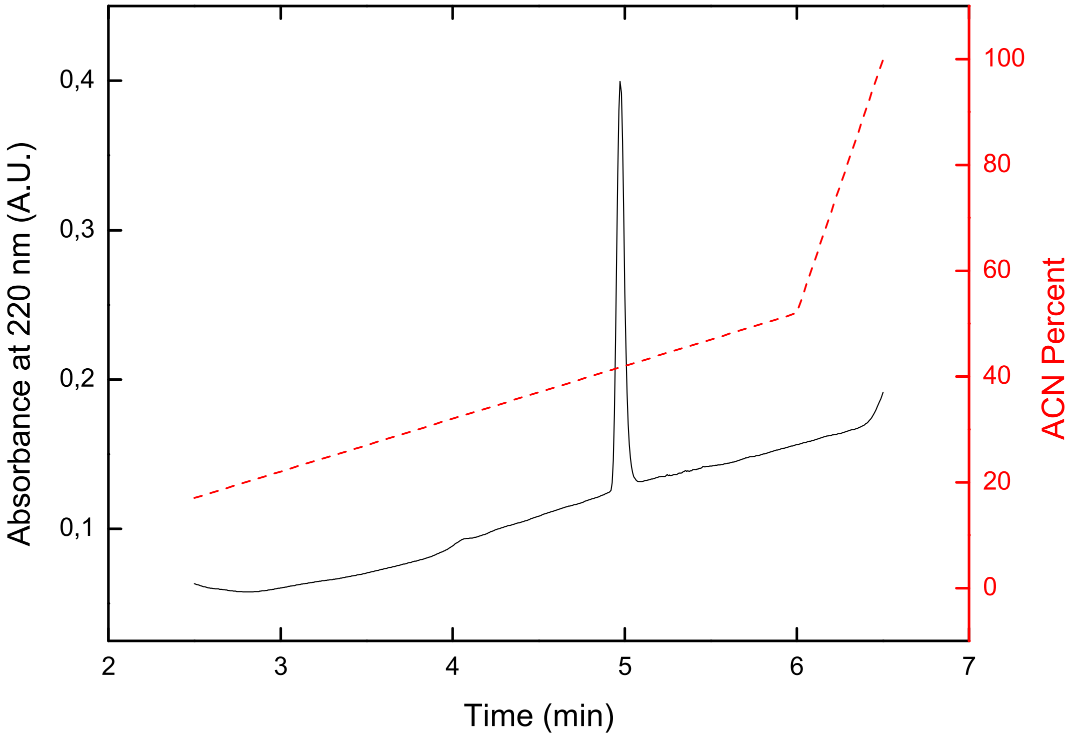

Figure S8. *m*/*z* spectrum of Ac-FHFWDDD-OH (**E**): theoretical MW_{mono} = 1022.366 Da.

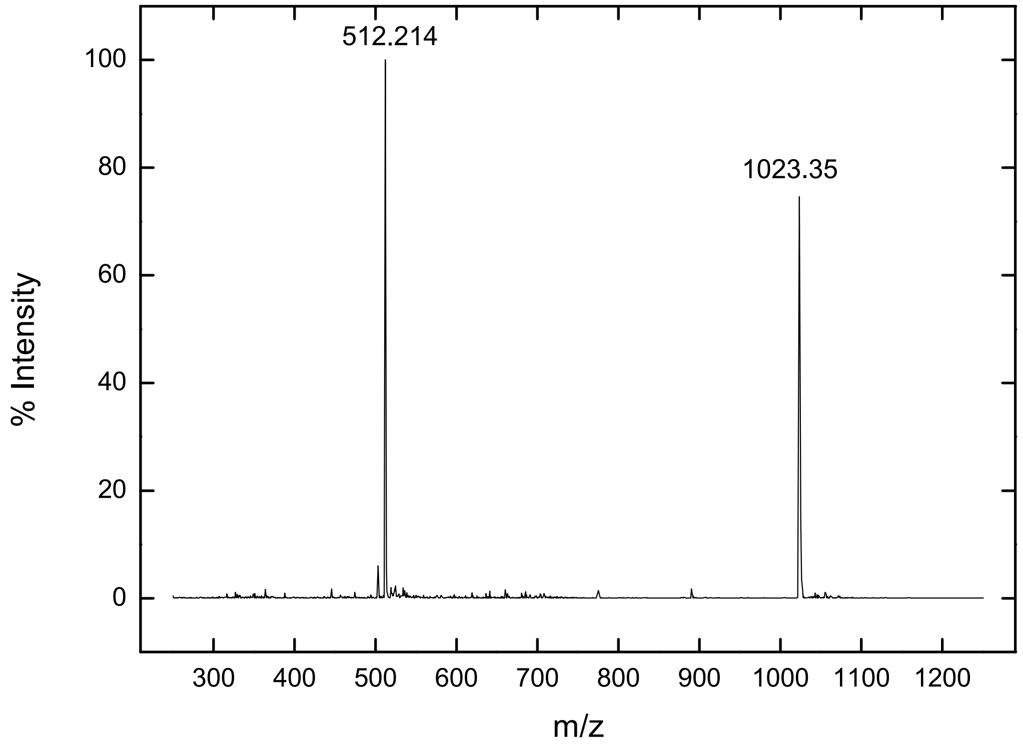

Figure S9. UHPLC chromatogram of Ac-LHLWDDD-OH (**F**). Gradient 2%–52% of ACN in 5 min with 0.1% HCOOH. Flow 0.4 mL/min. λ= 220 nm.

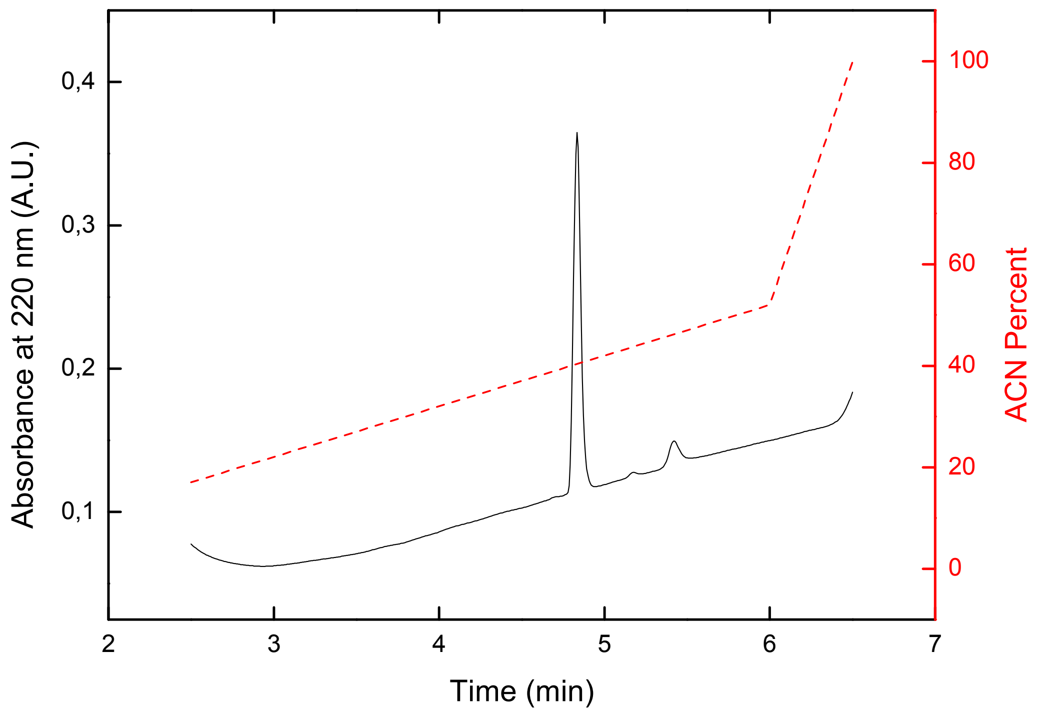

Figure S10. *m*/*z* spectrum of Ac-LHLWDDD-OH (**F**): theoretical MW_{mono} = 954.397 Da.

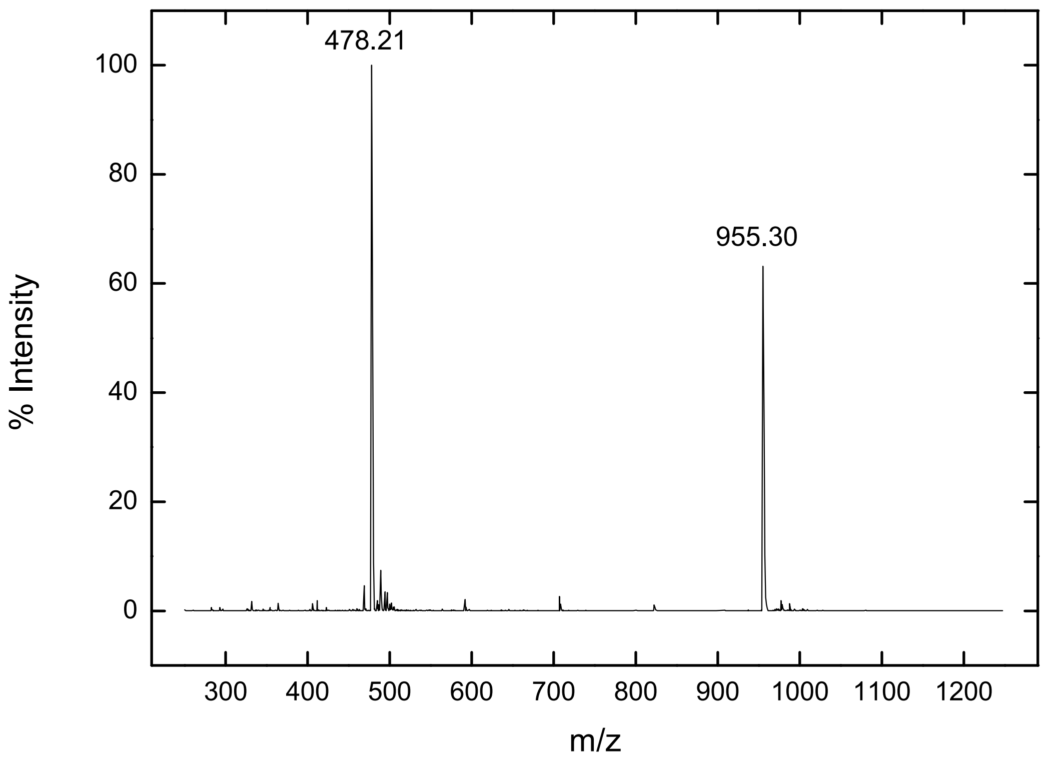

Figure S11. UHPLC chromatogram of Ac-SHSWDDD-OH (**G**). Gradient 2%–52% of ACN in 5 min with 0.1% HCOOH. Flow 0.4 mL/min. λ = 220 nm.

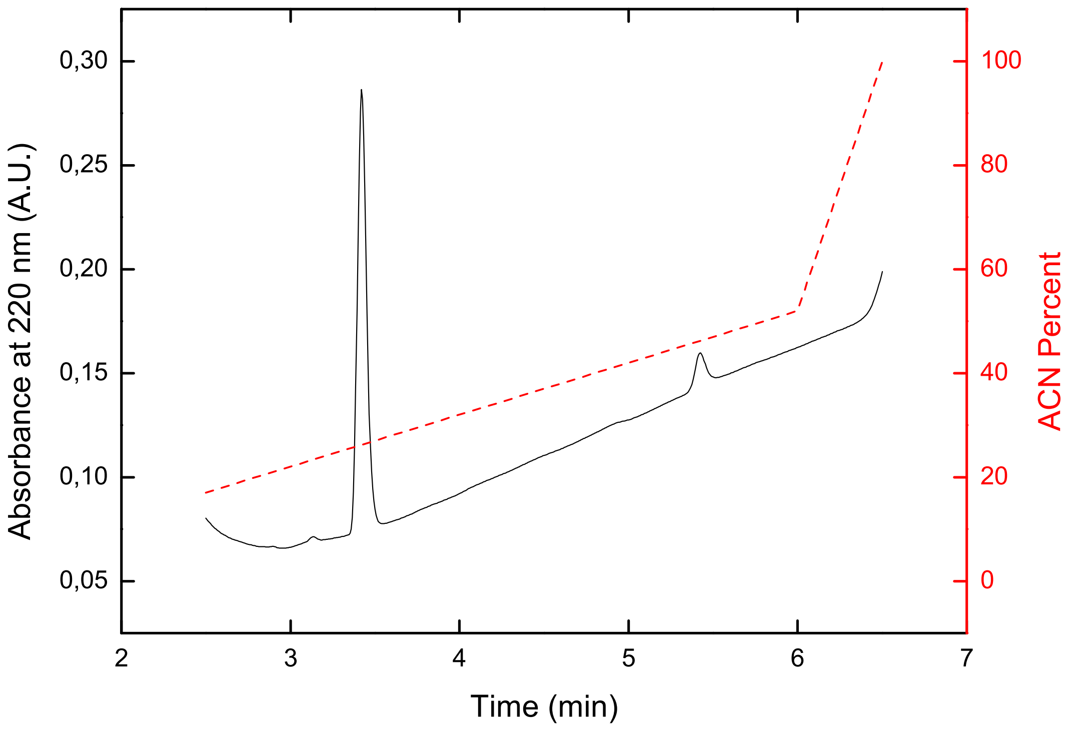

Figure S12. *m*/*z* spectrum of Ac-SHSWDDD-OH (**G**): theoretical MW_{mono} = 902.293 Da.

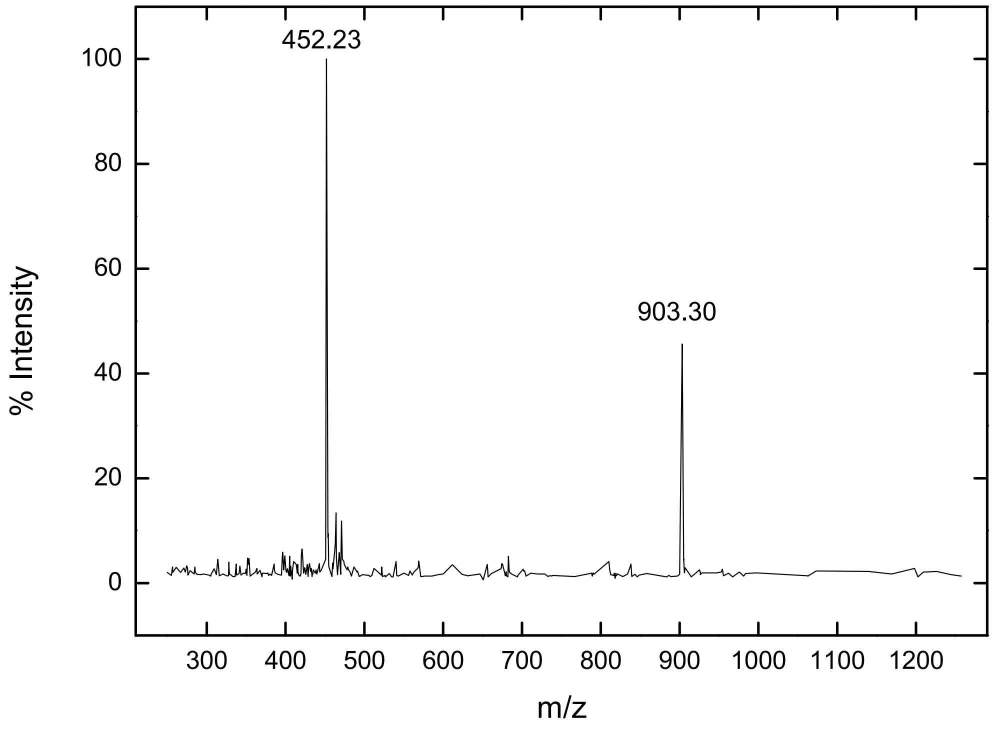

Figure S13. UHPLC chromatogram of Ac-YHYWDDD-OH (**H**). Gradient 2%–52% of ACN in 5 min with 0.1% HCOOH. Flow 0.4 mL/min. λ = 220 nm.

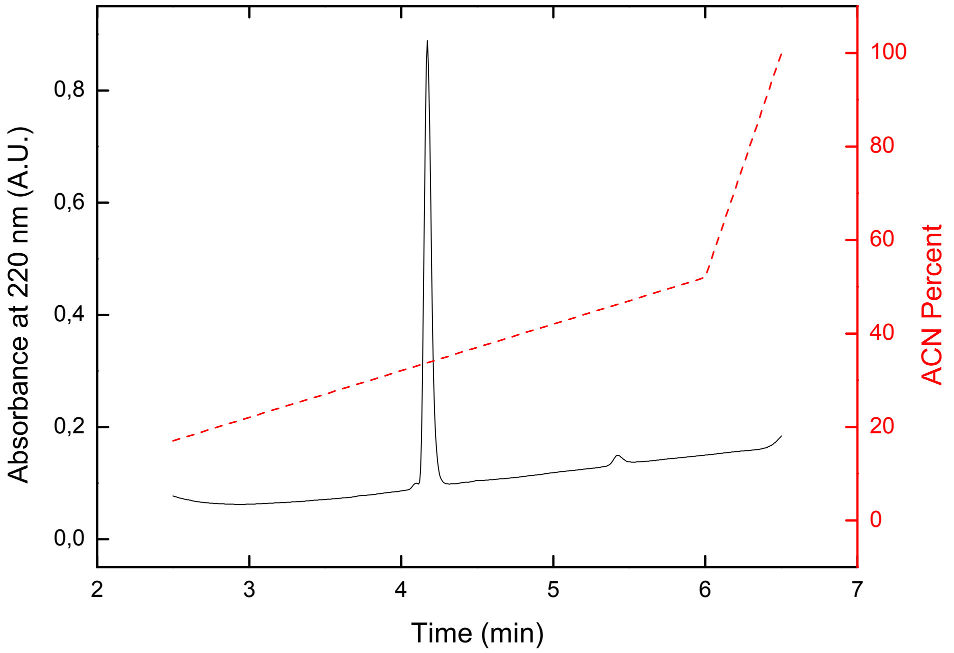

Figure S14. *m*/*z* spectrum of Ac-YHYWDDD-OH (**H**): theoretical MW_{mono} = 1054.356 Da.

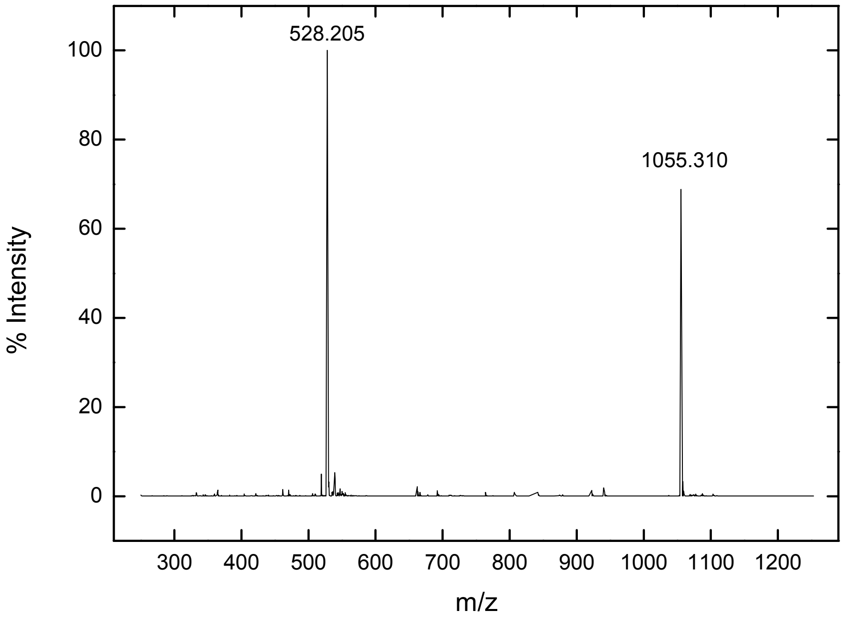

© 2013 by the authors; licensee MDPI, Basel, Switzerland. This article is an open access article distributed under the terms and conditions of the Creative Commons Attribution license (http://creativecommons.org/licenses/by/3.0/).
